# Lead optimization of 3-indoleacetonitrile identifies candidate compounds with improved anti-influenza activity and safety profiles

**DOI:** 10.3389/fmicb.2026.1904663

**Published:** 2026-08-12

**Authors:** Xianfeng Hui, Shihuan Ding, Bingqi Yan, Junlong Wang, Jingyun Zhang, Shuochen Xu, Rujing Zhang, Shuoxiang Gao, Tiesuo Zhao, Xiaowei Tian, Hui Wang

**Affiliations:** 1 Department of Immunology, School of Basic Medical Sciences, Henan Medical University, Xinxiang, China; 2 Henan Key Laboratory of Immunology and Targeted Drug, Henan Medical University, Xinxiang, China; 3 Xinxiang Engineering Technology Research Center of Immune Checkpoint Drug for Liver-Intestinal Tumors, Henan Medical University, Xinxiang, China; 4 Luoyang Central Hospital Affiliated to Henan Medical University, Luoyang, China; 5 Department of Pathogenic Biology, School of Basic Medical Sciences, Henan Medical University, Xinxiang, China; 6 Henan Collaborative Innovation Center of Molecular Diagnosis and Laboratory Medicine, School of Medical Technology, Henan Medical University, Xinxiang, China

**Keywords:** 3-indoleacetonitrile, antiviral therapy, indole derivatives, indole-3-ethanol, influenza A virus, small-molecule antivirals

## Abstract

**Introduction:**

Influenza A virus (IAV) remains a major respiratory pathogen that causes substantial morbidity, mortality, and socioeconomic burden worldwide. Although several antiviral agents are available for clinical use, their effectiveness is often compromised by the emergence of drug-resistant variants, limited therapeutic windows, and potential adverse effects. Small-molecule antivirals represent an important strategy for influenza treatment; however, improving antiviral efficacy while minimizing toxicity remains a major challenge in drug development. In this study, we sought to identify candidate low-toxicity derivatives structurally related to 3-indoleacetonitrile, a previously characterized compound with anti-influenza activity.

**Methods:**

Candidate compounds were selected through a computational screening strategy integrating structural similarity analysis, toxicity prediction, and drug-likeness assessment. Cytotoxicity was evaluated by CCK-8 assays and morphological observation in A549 cells. Antiviral activity was assessed using a recombinant luciferase-expressing PR8 influenza virus. Promising candidates were subsequently evaluated in a lethal influenza mouse model challenged with 10 × LD50 PR8 virus. Body weight changes, survival rates, lung pathology, viral burden, and serum AST/ALT levels were analyzed to determine antiviral efficacy and safety *in vivo*.

**Results:**

Four candidates—Indole-3-ethanol, Indole-3-acetamide, 3-Indoleacetic acid, and Acetohydroxamic acid—were identified. Cytotoxicity analyses revealed that Indole-3-ethanol and Indole-3-acetamide exhibited substantially lower toxicity than the parent compound 3-indoleacetonitrile, maintaining high cell viability at concentrations exceeding 640 μM. Morphological examination further confirmed their improved safety profiles. In antiviral assays, Indole-3-ethanol and Indole-3-acetamide demonstrated potent inhibition of IAV replication, while 3-Indoleacetic acid and Acetohydroxamic acid showed little or no detectable antiviral activity. *In vivo*, Indole-3-ethanol treatment alleviated body weight loss, delayed mortality, reduced pulmonary pathological injury, and significantly decreased viral loads in the lungs of PR8-infected mice. In addition, AST/ALT analysis indicated lower toxicity of Indole-3-ethanol and Indole-3-acetamide compared with 3-indoleacetonitrile.

**Conclusion:**

Structural optimization of the lead compound 3-indoleacetonitrile led to the identification of derivatives with improved antiviral activity and reduced toxicity. Among the compounds evaluated, Indole-3-ethanol exhibited the most favorable balance between efficacy and safety, demonstrating potent anti-influenza activity in both cell-based and animal models. These findings support the utility of computationally guided lead optimization and identify Indole-3-ethanol as a promising candidate for further development as an anti-influenza therapeutic.

## 1 Introduction

Influenza A virus (IAV) remains a major global public health threat, causing annual seasonal epidemics and occasional pandemics that result in substantial morbidity, mortality, and socioeconomic burden (Shah et al., 2022; Hui et al., 2022). According to the World Health Organization, seasonal influenza affects millions of individuals worldwide each year and is associated with hundreds of thousands of respiratory-related deaths. Certain populations, including pregnant women, older adults, young children, and immunocompromised individuals, are particularly susceptible to severe influenza-associated complications (CDC, n.d.). Despite the widespread use of vaccines, antigenic drift and shift continuously generate viral variants that compromise vaccine effectiveness, highlighting the ongoing need for effective antiviral interventions (Han et al., 2023).

Currently available anti-influenza drugs primarily include neuraminidase inhibitors, polymerase inhibitors, and cap-dependent endonuclease inhibitors (Hussain et al., 2025). Although these agents have demonstrated clinical benefits, their effectiveness is often constrained by the emergence of drug-resistant viral strains (Patel et al., 2023), limited therapeutic windows, and potential adverse effects. Furthermore, the number of approved anti-influenza drugs remains relatively limited, emphasizing the urgent need to identify antiviral agents with improved efficacy and safety profiles.

Small-molecule compounds have long served as an important source of antiviral therapeutics because of their favorable pharmacological properties, structural diversity, and potential for chemical optimization (Martin et al., 2023). In recent years, advances in artificial intelligence (AI) and machine learning technologies have revolutionized drug discovery by enabling rapid screening of large chemical libraries, prediction of biological activity, toxicity assessment, and optimization of lead compounds (Xu et al., 2022; Zhavoronkov et al., 2020). AI-assisted drug discovery has significantly reduced the time and cost associated with traditional screening approaches and has emerged as a powerful strategy for identifying novel antiviral candidates (Paul et al., 2021; Vamathevan et al., 2019).

Optimization of lead compounds represents a critical step in antiviral drug development. Many compounds with promising antiviral activity fail to progress because of unfavorable pharmacokinetic characteristics, poor solubility, or excessive toxicity (Mercorelli et al., 2022). Structural modification and derivative screening can improve therapeutic efficacy while simultaneously reducing toxicity, thereby enhancing the translational potential of candidate drugs (McMillan et al., 2023; Luan, 2025). Consequently, identifying structurally related analogs with improved antiviral potency and safety has become an important strategy in modern drug development.

Our previous studies demonstrated that 3-indoleacetonitrile exhibits broad-spectrum antiviral activity against influenza virus (Zhao et al., 2021) and SARS-CoV-2 (Hui et al., 2023) in both cell culture and animal models. Despite its promising antiviral efficacy, there remains scope for further optimization of its pharmacological properties to enhance its therapeutic potential and facilitate future development. To this end, we employed a computational screening strategy integrating structural similarity analysis, toxicity prediction, and drug-likeness assessment to identify candidate small molecules structurally related to 3-indoleacetonitrile. The selected compounds were subsequently subjected to systematic evaluation of their cytotoxicity, *in vitro* antiviral activity, and *in vivo* protective efficacy against influenza virus infection. Through this approach, we aimed to identify candidate compounds with improved safety profiles and potent antiviral activity, thereby providing promising candidates and a theoretical basis for the development of next-generation anti-influenza therapeutics.

## 2 Materials and methods

### 2.1 Compounds

Indole-3-acetamide (TopScience, T4834, Shanghai, China), Acetohydroxamic acid (TopScience, T1592, Shanghai, China), Indole-3-ethanol (TopScience, T4876, Shanghai, China), 3-indoleacetonitrile (TopScience, T8024, Shanghai, China), and 3-Indoleacetic acid (TopScience, T2O2791, Shanghai, China) were purchased from TopScience. All compounds were dissolved in dimethyl sulfoxide (DMSO) to prepare stock solutions. For *in vitro* assays, compounds were diluted in cell culture medium to the indicated concentrations. For *in vivo* administration, compounds were prepared in a vehicle containing 10% DMSO, 40% PEG300, 5% Tween 80, and 45% sterile water.

### 2.2 Cells and virus

The Madin-Darby Canine Kidney cells (MDCK) were maintained in Dulbecco’s modified Eagle’s medium (DMEM; Procellsystem, Wuhan, China), while human lung carcinoma epithelial cells (A549) were cultured in Ham’s F-12 medium (Procellsystem, Wuhan, China). All media were supplemented with 10% fetal bovine serum (FBS; Procellsystem, Wuhan, China) and cells were in cubated at 37 °C in a humidified atmosphere containing 5% CO₂. Influenza A virus, either the wild-type PR8 strain or the recombinant PR8 virus expressing luciferase (PR8-Luc), was propagated in 10-day-old embryonated chicken eggs, and viral titers were determined using the 50% tissue culture infectious dose (TCID_50_) assay (Cao et al., 2025).

### 2.3 Virus infection and antiviral evaluation of candidate compounds *in vitro*

The wild-type PR8 strain or the recombinant PR8 virus expressing luciferase (PR8-Luc) was diluted in serum-free medium to the desired viral titer. The diluted virus was then added to cells at approximately 90% confluence. After incubation for 60 min at 37 °C, the cells were gently washed twice with serum-free medium to remove unbound virus. Maintenance medium (1% serum) containing the candidate small molecule compounds or DMSO as a control was subsequently added for antiviral evaluation.

### 2.4 Cell counting Kit-8 assay

Cell viability was assessed using the Cell Counting Kit-8 (CCK-8, GlpBio Technology, Shanghai, China) according to the manufacturer’s protocol. Briefly, cells were seeded in 96-well plates at an appropriate density and allowed to attach overnight. After treatment with the small molecule compounds at the indicated concentrations for the specified time, 10 μL of CCK-8 reagent was added to each well, followed by incubation at 37 °C for 2 h. The absorbance at 450 nm was measured using a microplate reader. Cell viability was calculated relative to the DMSO-treated control and expressed as a percentage, following the protocol described in our previous report (Hui et al., 2023).

### 2.5 Reporter assays

To quantify influenza virus production in the culture supernatant, a viral luciferase assay was performed. Briefly, A549 cells in 96-well plates were infected with PR8-Luc virus and subsequently treated with the small molecule compounds or DMSO as a control. At the designated time points, culture supernatants were collected and centrifuged at 300 × g for 5 min to remove cell debris. Luciferase activity in the clarified supernatants was then measured using the Luciferase Assay System (Beyotime, Shanghai, China) according to the manufacturer’s instructions. The resulting luminescence directly reflected the viral yield in the supernatant, providing a quantitative assessment of the antiviral efficacy of the tested compounds.

### 2.6 Animal studies and ethics statement

Female 6–7-week-old BALB/c mice were used in the experiments and were purchased from Henan Skobes Biotechnology Co., Ltd. Animal experiments were approved by the Research Ethics Committee of Xinxiang Medical University, Henan, China (approval no. XYLL-2023-0127). All animal experiments were conducted in accordance with the recommendations of the Guide for the Care and Use of Laboratory Animals of the Research Ethics Committee, Henan Medical University, Henan, China. Prior to intranasal influenza virus infection, mice were anesthetized with isoflurane inhalation (3%–4% for induction and 1.5%–2% for maintenance in oxygen). At the experimental endpoint, mice were deeply anesthetized with isoflurane (5% in oxygen) until complete loss of reflex responses was achieved and were subsequently euthanized by cervical dislocation. Death was confirmed by cessation of respiration and heartbeat. All efforts were made to minimize animal suffering throughout the study.

To evaluate the effects of 3-indoleacetonitrile and other candidate small molecules on influenza virus infection and survival *in vivo*, mice were intraperitoneally administered 3-indoleacetonitrile (20 mg/kg) or vehicle control (10% DMSO in PBS containing PEG300 and Tween-80). Following infection, animals received daily intraperitoneal injections of 20 mg/kg of the indicated compounds or vehicle control from day 0 to day 4 post-infection. Body weight and survival were monitored daily for 14 days after infection. An additional group of uninfected mice receiving the same treatments was included to assess compound-associated effects on body weight in the absence of viral infection.

For lung viral burden analysis, lung tissues were collected at 4 days post-infection from three independent mice per group (*n* = 3) and subjected to quantitative the virus titers. Statistical analyses were performed based on this expanded dataset.

For histopathological evaluation, lung tissues were collected at 4 days post-infection. Representative H&E-stained sections from two mice per group were used to illustrate the typical pulmonary pathological changes observed in each experimental group. Histopathological observations were qualitative in nature and were not subjected to statistical analysis. All tissues were fixed in 4% paraformaldehyde for routine histological processing and hematoxylin and eosin (H&E) staining.

### 2.7 Serum biochemical analysis for toxicity evaluation

To comprehensively assess potential hepatic and renal toxicity induced by candidate compound treatment, serum samples were collected after 14 consecutive days of administration. Blood was collected and allowed to clot at room temperature, followed by centrifugation at 3,000 rpm for 10 min at 4 °C to obtain serum.

Serum aspartate aminotransferase (AST) and alanine aminotransferase (ALT) activities were measured using commercial assay kits (Abbkine, Wuhan, China) according to the manufacturer’s instructions, as previously described (Piao et al., 2023). Enzymatic activities were quantified using a microplate reader, and the AST/ALT ratio was calculated as an indicator of potential hepatic injury.

In addition, serum creatinine (CRE) and blood urea nitrogen (BUN) levels were measured using commercial assay kits (Abbkine, Wuhan, China) following the manufacturer’s protocols to evaluate renal function. All biochemical parameters were quantified using a microplate reader, and all samples were analyzed in duplicate.

### 2.8 Statistical analysis

Statistical analyses were performed using Student’s *t*-test for comparisons between two groups and two-way ANOVA for analyses involving multiple groups. Data were considered statistically significant at *p* < 0.05. Survival curves were generated using the Kaplan–Meier method and analyzed by the log-rank (Mantel–Cox) test. All statistical analyses were performed using GraphPad Prism software (version 8.0.2, GraphPad Software, San Diego, CA, USA). Comparisons between two groups were conducted using an unpaired two-tailed Student’s *t*-test.

## 3 Results

### 3.1 Computational screening and selection of candidate compounds structurally related to 3-indoleacetonitrile

**Figure 1:**
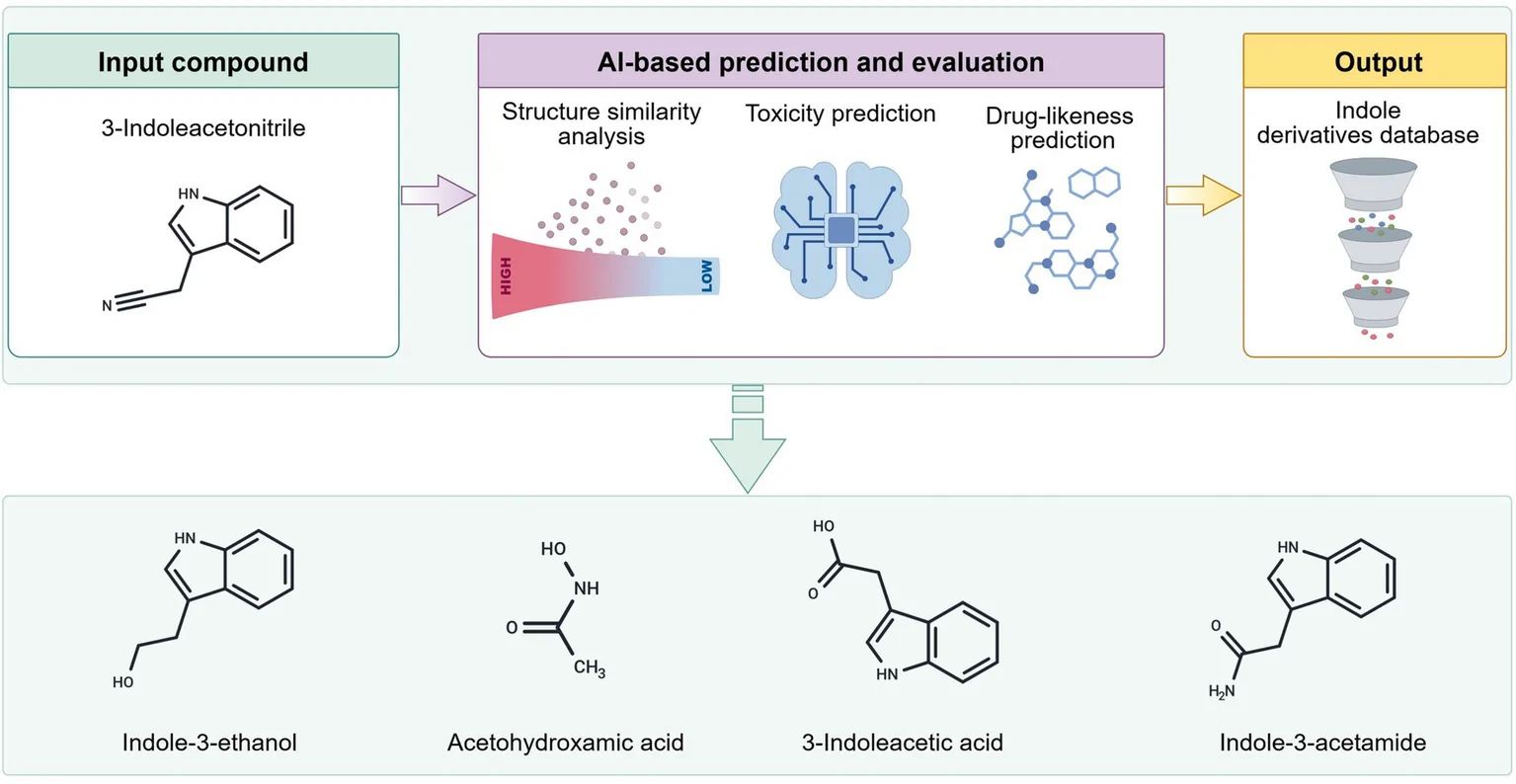
Computational screening and selection of candidate compounds structurally related to 3-indoleacetonitrile.

Based on artificial intelligence-assisted prediction, this study aimed to identify candidate small molecules structurally related to 3-indoleacetonitrile with improved antiviral activity and reduced cytotoxicity. By integrating structural similarity analysis, toxicity prediction, and drug-likeness evaluation, the AI-based screening pipeline identified four representative candidate compounds with potential anti-influenza activity, including Indole-3-ethanol, Acetohydroxamic acid, 3-Indoleacetic acid, and Indole-3-acetamide. These candidate molecules were subsequently selected for further *in vitro* and *in vivo* antiviral evaluation (Figure 1).

### 3.2 Cytotoxicity evaluation of candidate small molecules

**Figure 2:**
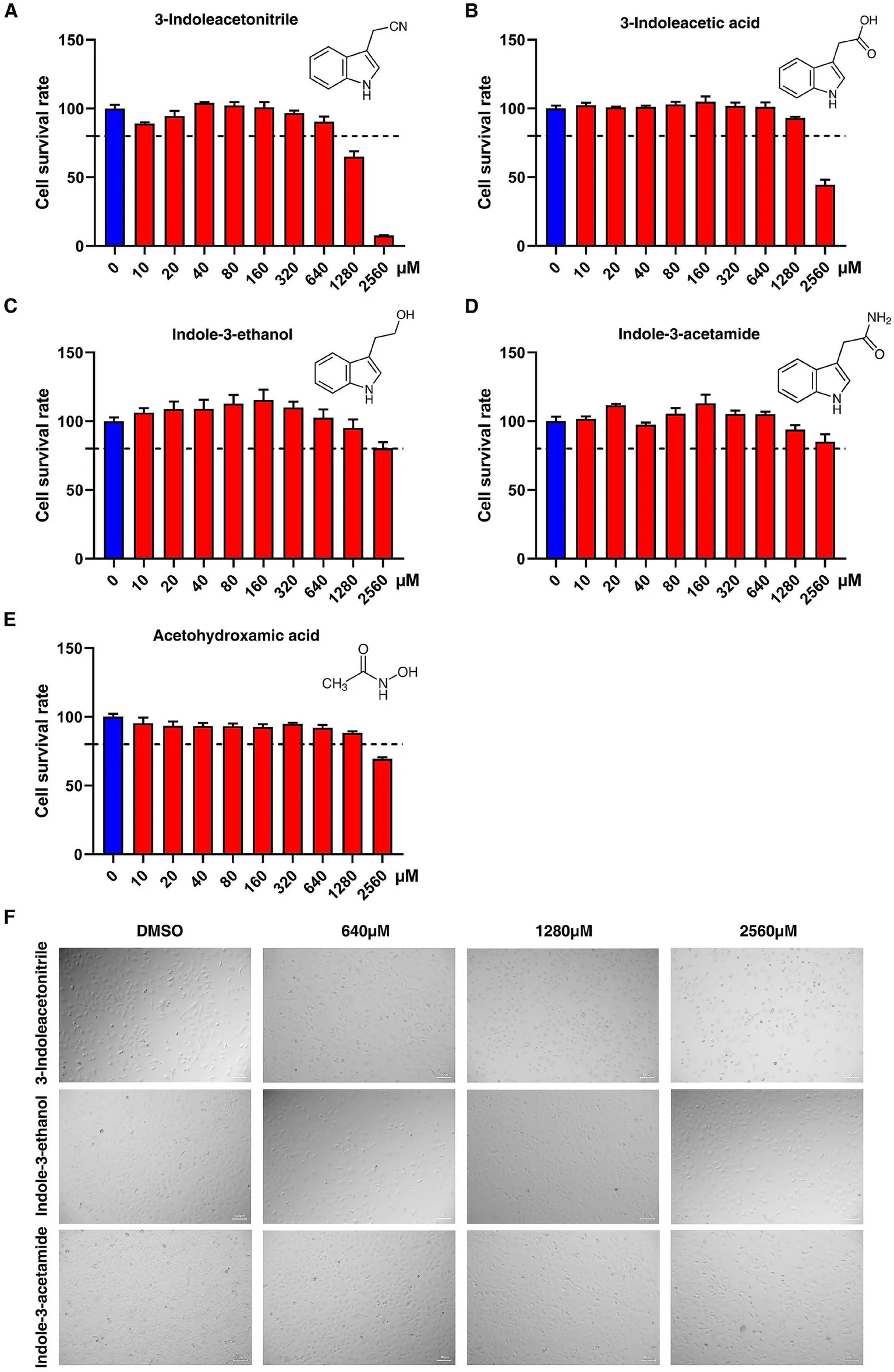
Indole-3-ethanol and Indole-3-acetamide exhibit lower cytotoxicity than 3-indoleacetonitrile. **(A–E)** A549 cells were treated with increasing concentrations of 3-indoleacetonitrile **(A)**, 3-Indoleacetic acid **(B)**, Indole-3-ethanol **(C)**, Indole-3-acetamide **(D)**, or Acetohydroxamic acid **(E)** for 24 h. Cell viability was subsequently determined using the CCK-8 assay. Indole-3-ethanol and Indole-3-acetamide exhibited markedly lower cytotoxicity than the parent compound 3-indoleacetonitrile and maintained high cell viability at elevated concentrations. **(F)** Representative microscopic images showing morphological changes in A549 cells following treatment with increasing concentrations of 3-indoleacetonitrile, Indole-3-ethanol, or Indole-3-acetamide for 24 h.

Prior to antiviral activity assessment, the cytotoxicity of the candidate small molecules was evaluated in vitro using the CCK-8 assay. The results demonstrated that, compared with 3-indoleacetonitrile, candidate compounds Indole-3-ethanol and Indole-3-acetamide exhibited markedly reduced cytotoxicity and maintained high cell viability even at relatively high concentrations (>640 μM; Figures 2A–E).

To further validate whether Indole-3-ethanol and Indole-3-acetamide exhibited lower cytotoxicity than 3-indoleacetonitrile, cells were treated with increasing concentrations of the three small molecules, and cellular morphology was examined microscopically after 24 h of exposure. The results demonstrated that treatment with 3-indoleacetonitrile induced evident morphological alterations at 1,280 μM, characterized by cell rounding and shrinkage. In contrast, cells treated with Indole-3-ethanol or Indole-3-acetamide maintained normal cellular morphology, with no obvious cytopathic changes observed even at concentrations up to 2,560 μM (Figure 2F). Collectively, these findings further support that Indole-3-ethanol and Indole-3-acetamide possess substantially lower cytotoxicity compared with the parent compound 3-indoleacetonitrile.

### 3.3 Evaluation of the *in vitro* antiviral activities of candidate small molecules

**Figure 3:**
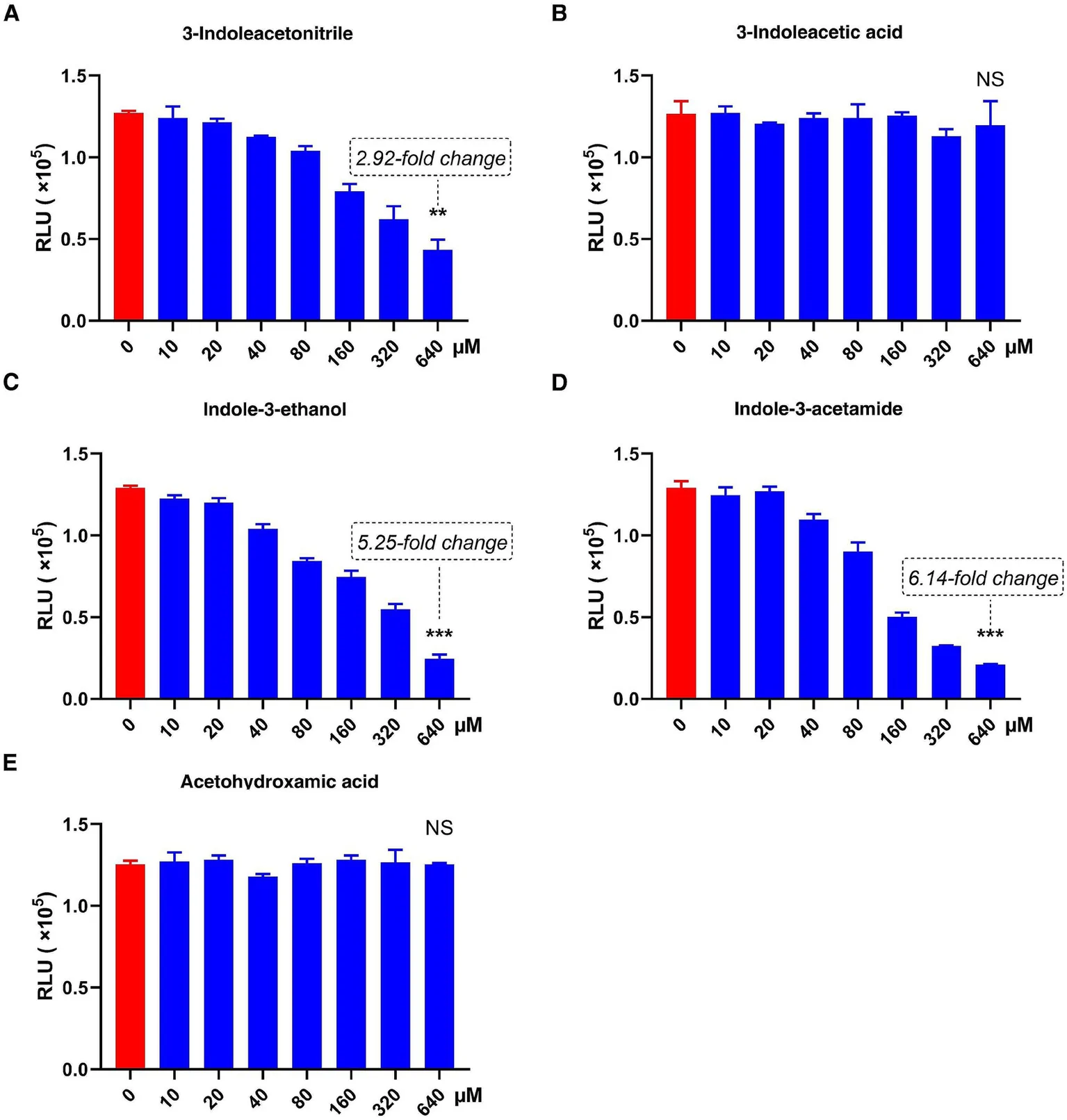
Indole-3-ethanol and indole-3-acetamide exhibit enhanced anti-influenza activity *in vitro.* **(A–E)** A549 cells were infected with the recombinant influenza A virus expressing luciferase (PR8-Luc) at an MOI of 0.05 and subsequently treated with increasing concentrations of 3-indoleacetonitrile **(A)**, 3-indoleacetic acid **(B)**, indole-3-ethanol **(C)**, indole-3-acetamide **(D)**, or acetohydroxamic acid **(E)**. At 36 h post-infection, culture supernatants were collected and luciferase activity was measured as an indirect indicator of viral replication and viral production. Statistical significance was determined using an unpaired *t*-test. *p < 0.05; **p < 0.01; ***p < 0.001; ****p < 0.0001; NS, not significant.

To further evaluate the in vitro antiviral activities of the candidate small molecules, a recombinant PR8 influenza virus expressing luciferase (PR8-Luc) was employed. This reporter system provides a surrogate readout of viral infection and replication-associated activity by measuring luciferase signals in infected cells, rather than directly quantifying infectious viral particles. The results showed that, at the maximum non-cytotoxic concentration of 640 μM, treatment with 3-indoleacetonitrile reduced luciferase intensity in the supernatant by approximately 2.92-fold compared with the 0 μM control group (Figure 3A). In contrast, neither 3-Indoleacetic acid (Figure 3B) nor Acetohydroxamic acid (Figure 3E) induced significant changes in luciferase activity at the same concentration, suggesting limited antiviral efficacy. Notably, Indole-3-ethanol (Figure 3C) and Indole-3-acetamide (Figure 3D) exhibited markedly enhanced antiviral activity, with luciferase intensity decreasing by approximately 5.25-fold and 6.14-fold, respectively, following treatment at 640 μM.

Collectively, these findings demonstrate that Indole-3-ethanol and Indole-3-acetamide possess substantially improved anti-influenza activity while maintaining lower cytotoxicity compared with the parent compound 3-indoleacetonitrile, highlighting their potential for further therapeutic development.

### 3.4 Indole-3-ethanol confers protective effects against influenza virus infection *in vivo*

**Figure 4:**
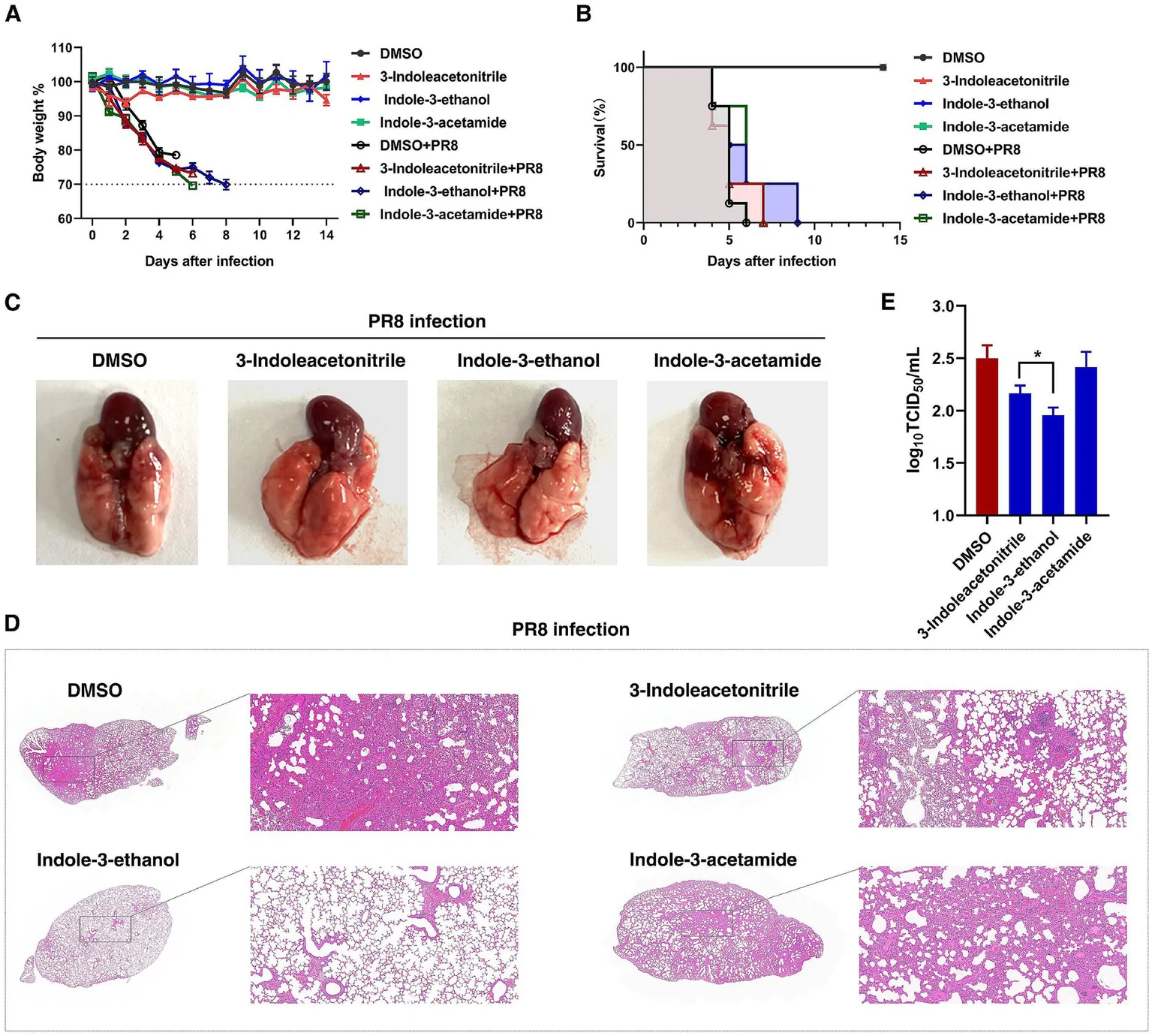
Indole-3-ethanol confers protective effects against influenza virus infection *in vivo* **(A)** Body weight changes of PR8-infected mice treated with DMSO, 3-indoleacetonitrile, Indole-3-ethanol, or Indole-3-acetamide following challenge with 10 × LD50 of PR8 virus. Body weight was monitored daily for 14 dpi. **(B)** Kaplan–Meier survival curves of PR8-infected mice receiving the indicated treatments. Survival was monitored for 14 dpi. **(C)** Representative gross pathological appearance of lungs collected from infected mice at 4 dpi. Pulmonary congestion and hemorrhagic lesions were evaluated among different treatment groups. **(D)** Histopathological analysis of lung tissues harvested at 4 dpi. Lung sections were stained with H&E and examined for inflammatory cell infiltration, alveolar destruction, and interstitial thickening. Representative images are shown. **(E)** Lung tissues were harvested at 4 dpi, homogenized, and infectious viral titers were determined by the TCID_50_ assay using MDCK cells. Statistical significance was determined using an unpaired *t*-test. *p < 0.05; **p < 0.01; ***p < 0.001; ****p < 0.0001; NS, not significant.

Given the promising antiviral activity of Indole-3-ethanol and Indole-3-acetamide observed *in vitro*, we next evaluated their protective effects against influenza virus infection in vivo. Mice were challenged with 10 × LD50 of PR8 virus and treated with 3-indoleacetonitrile, Indole-3-ethanol, or Indole-3-acetamide. Body weight loss, survival, and lung pathology were systematically assessed throughout the course of infection. Body weight monitoring from 0 to 14 days post-infection (dpi) revealed that, compared with the infected control group (DMSO + PR8), mice treated with Indole-3-ethanol exhibited a relatively pronounced protective effect, whereas the other candidate compounds showed only limited improvement in infection-induced weight loss (Figure 4A). Owing to the high-dose viral challenge used in this study, all groups experienced severe disease progression, with body weights declining to below 70% of their initial values or resulting in death by 9 dpi. Survival analysis further demonstrated that although Indole-3-ethanol treatment did not improve the final survival rate of infected mice, it delayed the onset of mortality and extended survival time under severe infection conditions, compared with both the infected control group and the 3-indoleacetonitrile-treated group (Figure 4B).

Gross pathological examination of the lungs showed that mice in the DMSO + PR8 group developed severe pulmonary congestion and hemorrhagic lesions. In contrast, lung damage was alleviated to varying degrees in both the 3-indoleacetonitrile + PR8 and Indole-3-ethanol + PR8 groups, with the most pronounced improvement observed in the Indole-3-ethanol-treated mice (Figure 4C). Histopathological analysis was consistent with these observations. Lung tissues from the DMSO + PR8 group exhibited typical features of viral pneumonia, including extensive alveolar destruction, massive inflammatory cell infiltration, and marked thickening of the pulmonary interstitium. In contrast, mice treated with Indole-3-ethanol displayed substantially reduced pathological damage, with relatively preserved alveolar architecture and significantly decreased inflammatory infiltration (Figure 4D). Further determination of infectious virus titers in lung tissues by TCID_50_ assay demonstrated that Indole-3-ethanol treatment significantly reduced pulmonary viral titers compared with the infected control group, indicating effective inhibition of influenza virus replication *in vivo* (Figure 4E). Collectively, these findings demonstrate that the selected candidate compounds exert antiviral effects *in vivo*, with Indole-3-ethanol showing the most consistent protective trends across multiple readouts, including body weight, pathology, and viral load, although no complete protection was achieved under the stringent 10 × LD50 challenge model.

### 3.5 Indole-3-ethanol exhibits favorable hepatic and renal safety following repeated administration

To further evaluate the in vivo safety profiles of the candidate compounds, uninfected mice were treated with DMSO, 3-indoleacetonitrile, Indole-3-ethanol, or Indole-3-acetamide, followed by serum biochemical and histopathological analyses.

**Figure 5:**
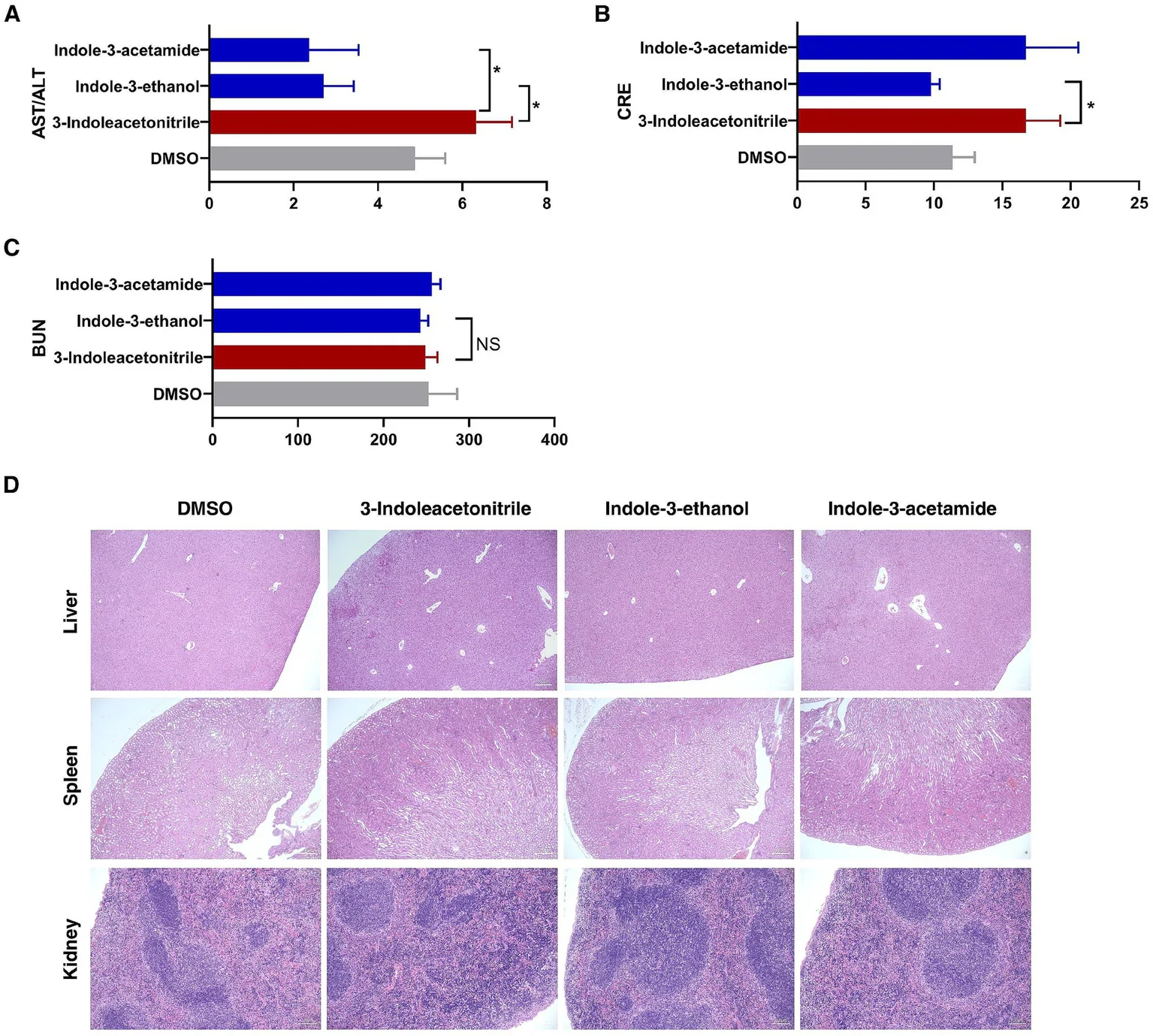
Evaluation of the in vivo safety profile of Indole-3-ethanol. Uninfected mice were administered DMSO, 3-indoleacetonitrile, Indole-3-ethanol, or Indole-3-acetamide (20 mg/kg) by intraperitoneal injection for 14 days. Serum and tissue samples were collected at the end of the treatment period for biochemical and histopathological analyses. **(A)** Serum AST/ALT ratios were determined to evaluate potential hepatotoxicity. **(B)** Serum creatinine (CRE) levels were measured to assess renal function. **(C)** Serum blood urea nitrogen (BUN) levels were measured as an additional indicator of renal function. Representative **(D)** H&E-stained sections of liver, kidney, and spleen tissues. Scale bars: 200 μm for the liver and kidney, and 100 μm for the spleen. Data are presented as the mean ± SD (*n* = 3 for serum biochemical analyses). Statistical significance was determined using an unpaired *t*-test. *p < 0.05; **p < 0.01; ***p < 0.001; ****p < 0.0001; NS, not significant.

Serum AST and ALT activities were first assessed to evaluate potential hepatotoxicity. Compared with the DMSO control group, mice treated with 3-indoleacetonitrile exhibited a modest increase in the AST/ALT ratio. In contrast, the AST/ALT ratios in the Indole-3-ethanol- and Indole-3-acetamide-treated groups were markedly lower than that of the 3-indoleacetonitrile group (Figure 5A). Although the AST/ALT ratios in these two groups were slightly lower than those in the DMSO control group, all values remained within the normal physiological range and therefore should not be interpreted as evidence of improved hepatic function. Rather, these findings indicate that neither compound induced detectable liver injury under the experimental conditions. Consistent with the serum biochemical data, histopathological examination revealed that Indole-3-ethanol markedly alleviated the hepatic histopathological alterations observed in the 3-indoleacetonitrile-treated group, further supporting its improved hepatic safety profile (Figure 5D).

To further evaluate potential renal toxicity, serum creatinine (CRE) and blood urea nitrogen (BUN) levels were measured. Compared with the DMSO control, 3-indoleacetonitrile treatment increased serum CRE levels. In contrast, serum CRE levels in the Indole-3-ethanol group were significantly lower than those in the 3-indoleacetonitrile group (Figure 5B). No significant differences in serum BUN levels were observed among the experimental groups (Figure 5C). Moreover, histopathological examination showed no obvious structural abnormalities in the kidneys of either treatment group (Figure 5D), suggesting that the elevated serum creatinine induced by 3-indoleacetonitrile was not accompanied by overt renal tissue damage. Together, these findings indicate that structural optimization to Indole-3-ethanol attenuated the mild renal functional alterations associated with the parent compound, resulting in an improved renal safety profile.

Histopathological examination of the spleen further demonstrated no obvious pathological abnormalities among the treatment groups (Figure 5D), indicating that neither compound produced detectable splenic toxicity under the experimental conditions.

Taken together, these biochemical and histopathological findings demonstrate that structural optimization of 3-indoleacetonitrile to Indole-3-ethanol substantially improved its *in vivo* safety profile by alleviating hepatic histopathological alterations and reducing the increase in serum creatinine, without inducing detectable histopathological abnormalities in the kidney or spleen. Combined with its potent antiviral activity observed both *in vitro* and in vivo, Indole-3-ethanol possesses an improved efficacy–safety profile and represents a promising lead compound for further anti-influenza drug development.

## 4 Discussion

Although several antiviral agents are available for the treatment of influenza, the continuous emergence of viral variants and antiviral resistance remains a challenge for effective disease control (Patel et al., 2023). In our previous work, 3-indoleacetonitrile exhibited antiviral activity against both influenza virus and SARS-CoV-2 (Hui et al., 2023). The present study extended these observations by evaluating structurally related candidate compounds. Among the compounds examined, Indole-3-ethanol emerged as the most promising candidate based on its antiviral efficacy and safety profile.

One notable observation was the substantial reduction in cytotoxicity following structural modification of the parent compound (Ju et al., 2022). Both Indole-3-ethanol and Indole-3-acetamide maintained high cellular viability over a broad concentration range and induced minimal morphological alterations compared with 3-indoleacetonitrile. These findings indicate that modest modifications within the indole scaffold can significantly affect cellular tolerance while preserving biological activity (Liu et al., 2023). Such improvements in safety are particularly important during lead optimization, as toxicity frequently limits the translational potential of otherwise active antiviral compounds.

In addition to their improved safety profiles, Indole-3-ethanol and Indole-3-acetamide demonstrated enhanced antiviral activity against influenza virus *in vitro*. Notably, antiviral activity was not uniformly retained among all structurally related molecules, as neither 3-Indoleacetic acid nor Acetohydroxamic acid substantially inhibited viral replication. This observation suggests that antiviral activity is closely associated with specific structural features rather than the presence of an indole backbone alone (Liu et al., 2023; Zhang et al., 2023). Further structure–activity relationship studies may help identify the molecular determinants responsible for antiviral efficacy and provide guidance for future chemical optimization.

Among the compounds tested, Indole-3-ethanol exhibited the most favorable performance *in vivo*. Treatment alleviated body weight loss, reduced lung pathological injury, and suppressed viral replication in infected animals. Although complete protection was not achieved under the highly stringent challenge conditions used in this study, the delay in disease progression and reduction in viral burden indicate a measurable therapeutic benefit (Murray et al., 2023). Considering that mice were challenged with 10 × LD50 PR8 virus, the degree of protection observed is likely to reflect genuine antiviral activity rather than nonspecific effects on disease symptoms (Dong et al., 2023). The observed in vivo efficacy should also be interpreted in the context of the dosing strategy employed in this study. The 20 mg/kg dose was selected based on our previous studies, in which this dose was established as the maximum safe dose of the parent compound, 3-indoleacetonitrile, in mice. Using the same dosing regimen allowed a direct comparison of antiviral efficacy and safety between the parent compound and its structurally optimized derivative, Indole-3-ethanol, under identical experimental conditions.

Safety evaluation further supported the potential of Indole-3-ethanol as a lead compound. Compared with its parent compound, 3-indoleacetonitrile, Indole-3-ethanol exhibited an improved safety profile, as evidenced by lower serum AST/ALT ratios, reduced serum creatinine levels, and attenuated hepatic histopathological alterations. Although treatment with 3-indoleacetonitrile resulted in elevated serum creatinine levels, no significant changes in serum BUN or renal histopathology were observed, suggesting that these renal effects were mild and not associated with overt structural damage. Likewise, no detectable histopathological abnormalities were observed in the kidney or spleen following Indole-3-ethanol treatment. Together with its lower cytotoxicity observed *in vitro*, these findings indicate that structural optimization not only enhanced antiviral efficacy but also improved the overall *in vivo* safety profile of Indole-3-ethanol.

Notably, although the AST/ALT ratios in the Indole-3-ethanol and Indole-3-acetamide groups were slightly lower than those in the DMSO control group, all values remained within the normal physiological range. Therefore, these differences should not be interpreted as evidence of enhanced hepatic function or hepatoprotective activity. Nevertheless, the biological significance of these modest biochemical differences relative to the DMSO control warrants further investigation through long-term toxicity studies and more comprehensive assessments of organ function.

The molecular mechanism responsible for the antiviral activity of Indole-3-ethanol remains unclear. Given its close structural relationship to 3-indoleacetonitrile, the two compounds may share common molecular features that contribute to their antiviral activity (Hui et al., 2023; Centofanti et al., 2022). However, the present study did not investigate the molecular mechanism of action, and therefore no conclusions can be drawn regarding the specific molecular targets or signaling pathways involved. Future studies will be required to identify the direct targets and elucidate the mechanism by which Indole-3-ethanol inhibits influenza virus replication.

It should be noted that the primary objective of this study was to identify and validate structurally optimized lead compounds with improved antiviral efficacy and safety relative to the parent molecule, rather than to establish a complete preclinical pharmacodynamic profile. Several limitations of this study should be considered. Only a small number of structurally related candidate compounds were evaluated, and broader chemical exploration may identify compounds with greater potency. In addition, although 3-indoleacetonitrile was included as an internal reference throughout the study, clinically approved anti-influenza drugs, such as oseltamivir or baloxavir, were not included as positive controls in the *in vitro* antiviral assays. Consequently, the antiviral activities of the tested compounds should be interpreted within the context of comparative lead optimization rather than as direct comparisons with currently available antiviral agents. Future studies incorporating licensed antivirals as external benchmarks will provide a more comprehensive assessment of the relative antiviral efficacy and translational potential of these compounds.

Furthermore, the *in vivo* efficacy and safety of Indole-3-ethanol were evaluated at a single dose (20 mg/kg), which was selected to enable direct comparison with the parent compound under identical experimental conditions. Consequently, the minimum effective dose, therapeutic window, and dose–response relationship of Indole-3-ethanol could not be determined in the present study. Future studies employing multiple dosing regimens will be necessary to define its pharmacodynamic characteristics and therapeutic index. Finally, in vivo efficacy was assessed using a single influenza virus strain, and its activity against other influenza subtypes or additional respiratory viruses remains to be determined.

In summary, structural optimization of 3-indoleacetonitrile led to the identification of candidate compounds with improved biological characteristics. Among them, Indole-3-ethanol displayed enhanced antiviral activity, reduced toxicity, and measurable protective efficacy *in vivo*, supporting its further development as a potential anti-influenza therapeutic.

## Data Availability

The raw data supporting the conclusions of this article will be made available by the authors, without undue reservation.
